# Effects of exposure to mother's and father's alcohol use on young children's normative perceptions of alcohol

**DOI:** 10.1111/acer.14902

**Published:** 2022-09-16

**Authors:** Megan Cook, Koen Smit, Carmen Voogt, Sandra Kuntsche, Emmanuel Kuntsche

**Affiliations:** ^1^ Centre for Alcohol Policy Research La Trobe University Melbourne Victoria Australia; ^2^ Trimbos Institute Netherlands Institute of Mental Health and Addiction Utrecht The Netherlands

**Keywords:** alcohol, children, drinking norms, eABT, exposure, fathers drinking, mothers drinking

## Abstract

**Background:**

While consumption of alcohol does not often begin until early adolescence, young children are highly capable of internalizing normative information through observational learning. We used a longitudinal multiple‐informant family study to examine the impact of exposure to mothers' and fathers' drinking on young children's normative perceptions of who drinks alcohol.

**Methods:**

Three hundred twenty‐nine children (4 to 6 years old at baseline [*M*_age_ 4.78 (SD = 0.725)], 51% girls) completed the Dutch electronic appropriate beverage task [eABT] where they attributed alcoholic beverages to a variety of persons depicted in an illustrated scenario. Their parents completed an online survey that included information on alcohol use and exposure.

**Results:**

Children more frequently exposed to their mothers' drinking provided females shown in the eABT illustrations with alcohol significantly more often than children less frequently exposed to mothers' drinking. There was no effect of mother's exposure on providing males in the eABT with alcoholic beverages. Similarly, children more frequently exposed to their fathers' drinking provided fathers with alcoholic beverages significantly more often than children less frequently exposed to their fathers' drinking. There was no effect of father's exposure on providing the females with alcoholic beverages, nor was there an effect of father's exposure on providing “other males” with alcohol. These patterns held after adjusting for age and sex.

**Conclusions:**

This study demonstrates that there are gender‐specific effects of exposure to parents' (particularly mothers') drinking on young children's perceptions of person‐specific drinking norms. The findings provide unique evidence in a young population group of effects on an understudied dimension of alcohol‐related perceptions with implications for future drinking behavior.

## Graphical abstract

329 children (four‐to‐eight years old) completed the Dutch electronic Appropriate Beverage Task [eABT], while parents completed a survey. Children more frequently exposed to their mothers’ drinking provided females in the eABT significantly more often with alcohol (β = 1.302, SE = .92***), while children more frequently exposed to their fathers’ drinking provided males in the eABT significantly more often with alcohol (β = .663, SE = .249**) than children less frequently exposed to mothers’ and fathers drinking respectively. Results demonstrate the gender‐specific effect of exposure to parents drinking.

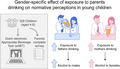

## INTRODUCTION

Although consumption of alcohol does not often begin until adolescence, young children have been shown to be highly capable of internalizing normative information through observational learning (Bandura, 1977), thereby holding an extensive amount of knowledge on alcohol‐related cognitions and adults' drinking practices from a young age, that is, under 8 years (Cook, Kuntsche, Smit, et al., 2021; Kuntsche et al., 2016). When consumption is initiated, it is based on years of this observational learning alongside the physical and social contexts in which children live, alcohol's availability, price, and marketing. Although there are a variety of potential sources, when children are at a young age parents play a primary role in observational learning and modeling (Bandura, 1977).

In terms of intergenerational transmission of alcohol‐related cognitions, recent research has demonstrated that what is important is not how much parents drink in general, but rather when they drink in the *presence* of children (Smit et al., 2019). For example, some parents may consume alcohol frequently but only when out with adult friends or when children are in bed. Thus, children have zero exposure to parents drinking. By comparison, other parents may consume alcohol less frequently, but anytime they do consume, it is in the presence of their children. Smit and colleagues demonstrated the importance of this association for young adolescent's alcohol expectancies, finding exposure mediated the association between parental drinking and preteens alcohol use (Smit et al., 2018, 2019, 2020). However, research considering the role of exposure to parents drinking on young children's normative perceptions is lacking. This is despite young children being shown to hold extensive normative perceptions, for example, person‐specific norms, with males thought to consume more than females (Kuntsche et al., 2016). What is more, norms play a role in regulating young people's drinking, with drinking norms found to predict heavy^1^ and problem drinking^2^ in late adolescence (17‐ to 19‐years; Voogt et al., 2013). Drinking norms have also shown to be associated with drinking behaviors over time (Brody et al., 2000; Payne et al., 2016; Zucker et al., 2008). To fill this gap, we investigate the influence of exposure to mothers and fathers drinking on children's normative perceptions of who usually drinks alcohol.

Following social learning theory (Bandura, 1977) and the cognitive model of intergenerational transference (Campbell & Oei, 2010), learning about alcohol at young ages primarily occurs through the observation of models of the behavior, most commonly parents. However, findings relating to the impact of parents' alcohol use on children's alcohol‐related perceptions are far from straightforward. Cross‐sectional evidence has shown parental alcohol use to be both positively related to children's acquisition of alcohol‐related knowledge (Kuntsche & Kuntsche, 2019; Mennella & Garcia, 2000), norms (Dalton et al., 2005) and expectancies (Donovan et al., 2009; Epstein et al., 2008; Kuntsche & Kuntsche, 2018; Waddell et al., 2020), but also not to have any effect on children's knowledge of alcohol (Hahn et al., 2000; Jahoda et al., 1980). Variations in the cognitive dimension being examined, difference in the age of the child, cultural differences, differences in measures of alcohol use or the ability to measure mothers and fathers separately may be behind the varied findings reported thus far. For example, among those 12‐to‐15‐year‐olds, fathers drinking has been found to have a larger effect on offspring's alcohol‐related cognitions than mothers (Smit et al., 2019), arguably because they consume more than women (Van Laar et al., 2021). Similarly, in a study among those 3‐to 6‐year‐olds, fathers drinking was also found to have a larger effect than mothers on children's expectancies (Kuntsche & Kuntsche, 2018). In comparison with looking at whether there is an effect between cognitions and use, exploring how exposure may affect cognitive dimensions has been little studied. Thus, more work breaking down the potential associations is needed, for example, any differences in the influence of mothers' and fathers' exposure or differences in exposures influence on the acquisition of alcohol‐related cognitions.

Apart from work by Smit et al. (2018, 2019, 2020) on the role of exposure to parental drinking on adolescent alcohol expectancies mentioned above, few other studies have examined exposure among young children. Moreover, and to the best of our knowledge, no previous studies have examined exposures influence on young children's normative perceptions. This is despite the fundamental role norms play as “drinking rules” (i.e., collective standards which guide behavior), which is reflected in their strong presence in young children well before they first consume. For example, recent work from the Netherlands has shown that children as young as four have normative perceptions about who drinks alcohol and where alcohol is consumed, which gets stronger over time (Cook, Kuntsche, Smit, et al., 2021; Cook, Smit, et al., 2022; Voogt et al., 2020). Thus, there is considerable scope and impetus to investigate the origins of normative perceptions, starting with parental exposure which has been shown to play a key role for other cognitive dimensions among other population groups (i.e., expectancies among adolescents (Smit et al., 2019)).

This study used a longitudinal multiple‐informant family design with data collected using the Dutch electronic appropriate beverage task (eABT; Voogt et al., 2017) and corresponding parental survey to investigate intergenerational exposure on normative perceptions. Specifically, we are investigating the effect of exposure to mothers and father drinking on children's perceptions of who is more likely to drink alcohol across the variety of persons presented in the task. To do so, we ran three models.

1. In the first model, we examined exposure to mothers and fathers drinking on children's attributions of alcohol to the females or the males depicted in the eABT, that is overall person‐specific drinking norms. We hypothesize that exposure from both mothers and fathers drinking will affect children's overall normative perceptions (i.e., we expect exposure to both mothers and fathers drinking to be positively associated with children's alcohol‐related normative perceptions).
2. In the second model, we examined the influence of exposure to parental drinking across the people depicted in the task. This was to examine whether the association was different dependent on the role of the person in the picture, for example, whether exposure to mothers drinking influenced children's attributions of alcohol to the mothers only, or whether it was dependent upon the gender of the person depicted in the eABT. For example, the more children are exposed to father's drinking, the more they will provide male persons in the eABT scenarios with any alcoholic drink but not female persons. Based on social learning theory (Bandura, 1977), which suggests that replication of observed behavior is more likely when the model is “similar” to the observer, for example of the same gender, we hypothesis that exposure to mothers drinking will affect attributions to females, and exposure to fathers drinking will affect attributions to males.
3. In the final model, we will test whether these associations will remain significant when adjusting for children's age and sex.

## METHODS

### Sample and procedure

Data were drawn from a three‐wave multi‐informant longitudinal family study including children aged four to six at baseline [*M*_age_ 4.78 (SD = 0.725)] and their parents in the Netherlands. The purpose of this family study was to examine precursors of alcohol consumption rooted in childhood (specifically alcohol‐related cognitions) and to consider intergenerational transference of these cognitions. Primary schools (*n* = 831) were contacted in five regions in the Netherlands (Gelderland, Flevoland, Groningen, Zeeland, and Zuid‐Holland), with those who agreed to participate (*n* = 92) asked to distribute letters of invitation to obtain parental consent for first‐ and second‐grade students' participation (more details can be found in Voogt et al., 2017, 2020).

Children (51% girls) completed the Dutch eABT on tablet computers during home visits in 2015, 2016, and 2017 (lasting approximately 40 min). Retention rates were 97.9% (*n* = 322) at T2 and 97.6% (*n* = 321) at T3. A majority of participants lived with both their mother and father (92%), most parents reported being married or in a registered partnership (75%) and reported being of Dutch nationality (96% of mothers and 99% of fathers, 98% children).

**FIGURE 1:**
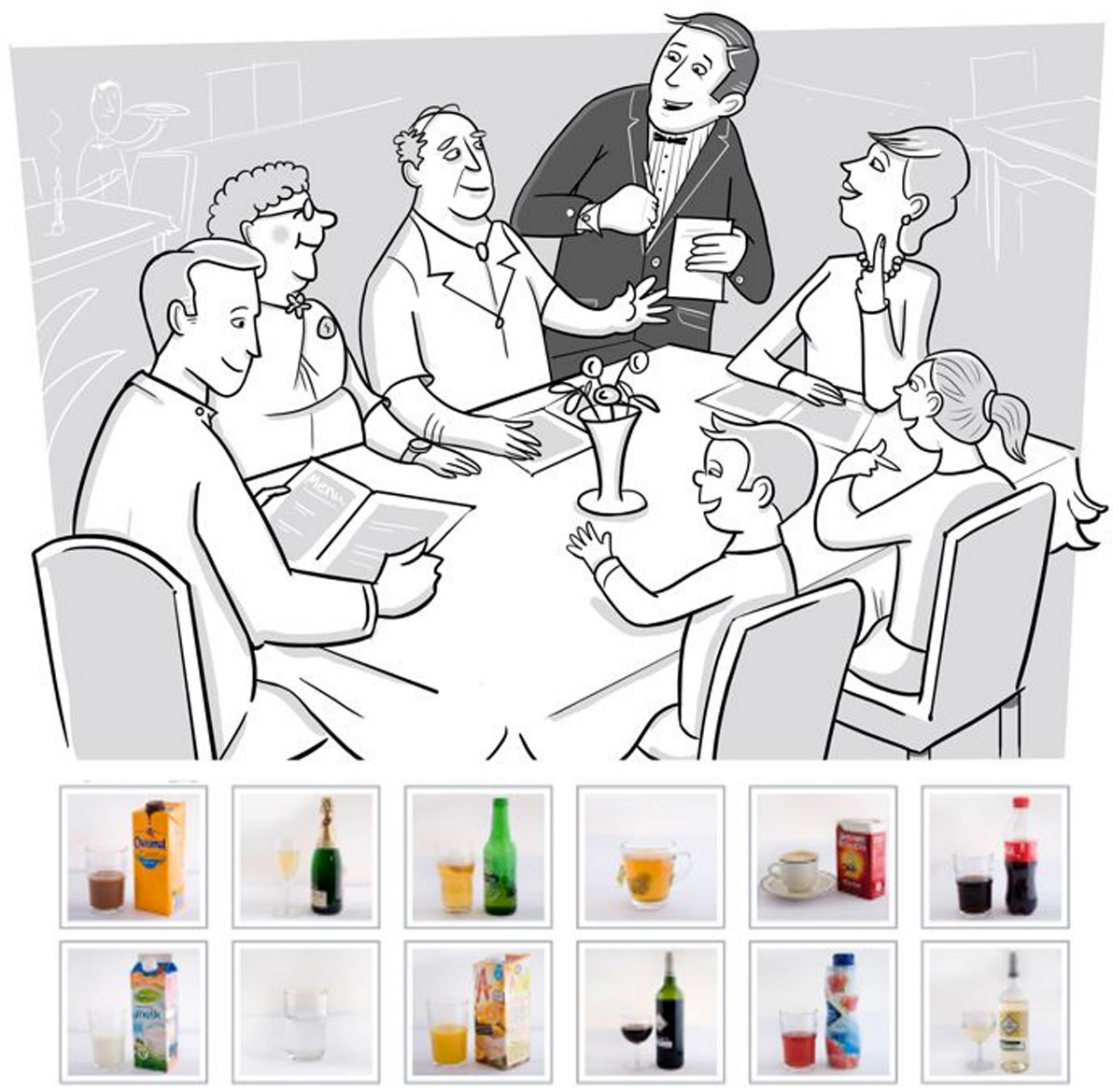
Restaurant situation from the Dutch electronic appropriate beverage task (one of five common illustrated situations depicted). The five common drinking situations were—party, Christmas, restaurant, barbeque, and terrace. The eABT includes four alcoholic (beer, champagne, red wine, and white wine) and eight nonalcoholic beverages (chocolate milk, cola, coffee, lemonade, milk, orange juice, tea, and water)

The task presents children with a range of illustrated situations (see Figure 1 for an example) depicting a range of individuals. Participants are asked to choose one of 12 beverages (four alcoholic and eight nonalcoholic) underneath the illustration to “attribute” to the individual at whom an arrow is pointing; to do so, they tap on the picture of the beverage they think the person in the illustrated situation has been drinking. Illustrated situations are presented one at a time and in a random order. A beverage is attributed to each person in the illustrated situation before moving onto the next situation (answers are stored in a secure database). While adults and children are depicted in the task, the following analysis only includes beverages attributed to the adults, in situations in which it is common to drink (Voogt et al., 2017, 2020).

While children completed the eABT with the assistance of a member of the research team, parents (both mothers and fathers) completed an online survey. There was no time limit on completion, and children were provided with a practice situation (e.g., providing food to farm animals) to familiarize them with the procedure. At the end of each home visit, a small present (e.g., pencil) was provided to the child and one parent received a gift coupon of 10€ as an incentive. Approval was obtained from the Ethical Committee of the Faculty of Social Sciences from Radboud University (ECSW2014‐2411‐272).

### Measures—children's eABT

#### Persons depicted in the illustrations

Each illustrated situation depicted a range of persons in a situational context (see Figure 1 for an example). Researchers recorded the gender and role of the persons in the illustrations and then grouped the persons for analysis as follows—females (all females depicted), males (all males depicted), mothers, fathers, other females (including female friends and grandmothers), and other males (including male friends and grandfathers). We take the association between the beverage attributed (alcoholic or nonalcoholic) and the person in the illustrated situation as evidence of a child's perception of a normative relationship.

#### Alcoholic beverages

When completing the task, children were asked to tap on the picture of the beverage that they believed the person in the illustrated situation, as indicated by an arrow, was drinking. These were the children's “attributions.” Given that beverage‐specific explorations have been reported previously using this sample (Cook, Kuntsche, Smit, et al., 2021), the focus of this paper is on children's perceptions of who is more likely to drink alcohol overall. Attributions of any of the four alcoholic beverages (of 12 total beverages) to the persons in the illustrated situations were coded into a dichotomous variable indicating alcohol attributions (1) or not (0) at each of the three time points (from which we created an overall mean score—see analytic strategy section for more details).

#### Participant sex

Participant sex was recorded by the parent/legal guardian, and coded as 0 = boys, 1 = girls.

#### Age at baseline

Age was recorded at baseline and coded as the subgroups—4, 5, and 6 years.

### Measures—parental questionnaire

#### Parental alcohol use frequency

Parents' alcohol use was assessed using the question: “How often did you drink alcohol in the past four weeks?.” The response categories were recoded into the number of days on which alcohol was consumed per month as 0 = “no consumption in the past four weeks,” 2 = “one to three drinks per month,” 4.25 = “one to two drinks per week,” 14.875 = “three to four drinks per week,” 23.375 = “five to six drinks per week,” and 30 = “drinking every day.”

#### Exposure to parental alcohol use

Parents responded to a questionnaire on their consumption in nine drinking situations (this ranking was based on previous research with the same sample, Voogt et al., 2020). These situations were dinner, Christmas, party, watching tv, barbeque, terrace restaurant, and camping. The items asked participants how often they drank alcohol in the situations that corresponded to the family‐related situations included in the eABT which the children completed. The response categories ranged from 0 = “never” to 4 = “always*”.* This information was reported by both mothers and fathers. We created a mean score across the nine situations for mothers and fathers separately.

### Analytic strategy

Of the 329 children who completed the task, 24 (7.3%) had missing data on the variables of interest from both parents at one or more waves of data collection and four had missing data from both parents at all three waves. Descriptive analysis, including independent samples *t*‐tests and standardized mean effect sizes (Cohen's *D*), were first conducted in SPSS and are presented in Table 1, then regression analysis was conducted using the Mplus statistical software (Muthén & Muthén, 1998‐2020) to examine intergenerational exposure on normative perceptions. Missing data were accounted for using the full‐information maximum likelihood option in Mplus. Given we were not interested in changes over time, we created a mean score of the data from the three annual time points (collected 2015, 2016, and 2017, meaning children were between 4‐ and 8‐year‐olds).

**TABLE 1:** Means (SD) of parent's alcohol‐related behaviors and children's exposure separate for fathers and mothers

	Fathers	Mothers	*t*‐value	Cohen's *D*
Parents				
Alcohol use frequency^a^	7.20 (7.80)	4.36 (6.54)	8.312***	0.40
Alcohol exposure	1.98 (0.798)	1.52 (0.903)	11.66***	0.54
Children				
Girls' exposure to parents drinking	2.06 (0.814)	1.58 (0.864)	8.68***	0.57
Boys' exposure to parents drinking	1.91 (0.775)	1.45 (0.939)	7.84***	0.53

*** *p* < 0.001.

^a^ Consumption refers to the frequency of consumption in the past 4 weeks.

Three ordinary least‐squares (linear) regression models were estimated. In the first model, children's attributions of alcohol to the females and the males depicted in the eABT (this is the mean score across the three time points) were regressed on exposure to mothers and fathers drinking, respectively. This allowed us to examine overall person‐specific drinking norms (hypothesis 1). In the second model, we examined whether exposure to mothers drinking was linked to children's attributions of alcohol to the mothers, fathers, other females, and other males depicted in the eABT, and again the same for fathers (hypothesis 2). In the third model, we examined whether the emerging association remained significant when adjusting for children's age and sex.

## RESULTS

Descriptive results show that on average fathers had consumed alcohol about nine times in the last 4 weeks, about three and a half drinks more than mothers (see Table 1). Mothers drinking in the last 4 weeks was moderately correlated with fathers drinking in the past 4 weeks (*r* = 0.300, *p* = <0.001). Girls were exposed to fathers drinking in two (2.06) situations on average, and mothers drinking in 1.5 situations. In terms of the mean level of mothers' and fathers' exposure, independent samples *t*‐test indicated that fathers were more likely to report drinking in situations that expose children to alcohol (i.e., when at a restaurant), than mothers. Exposure to mothers drinking was moderately correlated with exposure to fathers drinking (*r* = 0.389, *p* = <0.001). Descriptive results also show that children attributed alcohol to the females in the illustrations 51% of the time, mothers 35%, other females 16% (including female friends and grandmothers), males 89%, fathers 62%, and other males 26% (including male friends and grandfathers).

### Regression model

Regression results presented in Table 2 show that children more often exposed to their mothers' drinking provided the females in the illustrations significantly more often with alcoholic beverages, than children less frequently exposed to mothers drinking. There was no effect of exposure to mother's drinking on providing the males in the illustrations with alcoholic beverages. Similarly, children more frequently exposed to their fathers' drinking, provided the males in the illustrations significantly more often with alcoholic beverages, than children less frequently exposed to fathers drinking. Again, there was no effect of exposure to father's drinking on providing the females in the illustrations with alcoholic beverages.

**TABLE 2:** Exposure to fathers and mothers drinking on children's attributions to the females and males in the illustration (unstandardized regressions coefficients and standard errors in brackets)

Model 1	Persons depicted in the illustrations	
	Females	Males
	β (SE)	β (SE)
Exposure to mothers' drinking	1.302 (0.92)***	−0.166 (0.205)
Exposure to fathers' drinking	−0.016 (0.221)	0.663 (0.249)**

**p < 0.01; ***p < 0.001.

In Table 3, we found that exposure to mothers' drinking was associated with children's attributions of alcohol to all females depicted in the task, while exposure to fathers' drinking was significantly associated with children's attributions of alcohol only to fathers in the task and not the other males (Table 3). We also found girls attributed less alcohol to mothers than boys but not to any other adult person depicted in the eABT. Older children attributed more alcohol to fathers and other adult males (see Table S2 for further analyses of sex effects).

**TABLE 3:** Mothers' and fathers' exposure on children's attributions to the mothers, fathers, and other males and females in the illustration with age as a covariate (unstandardized regressions coefficients and standard errors in brackets)

	Mothers	Fathers	Other females	Other males
	β (SE)	β (SE)	β (SE)	β (SE)
Model 2				
Exposure to mothers' drinking	0.994 (0.143)***	0.007 (0.171)	0.307 (0.070)***	−0.023 (0.076)
Exposure to fathers' drinking	−0.137 (0.158)	0.557 (0.188)**	−0.029 (0.071)	0.105 (0.090)
Model 3				
Exposure to mothers' drinking	1.028 (0.142)***	0.044 (0.170)	0.320 (0.072)***	−0.002 (0.075)
Exposure to fathers' drinking	−0.123 (0.156)	0.488 (0.189)*	−0.037 (0.071)	0.057 (0.089)
Sex	−0.747 (0.252)**	0.165 (0.296)	−0.123 (0.119)	0.215 (0.134)
Age	0.130 (0.161)	0.488 (0.197)*	0.103 (0.083)	0.310 (0.088)***

*Note*: Sex coded 0 = boys, 1 = girls.

The category “females” was broken down into “mothers and “other females” including female friends and grandmothers and the category “males” was broken down into “fathers” and “other males” including male friends and grandfathers, to examine whether attributions to one role were driving the attributions for the gender found. Further examination of the significant sex findings is provided in the supplementary material (Table S2).

*p < 0.05; **p < 0.01; ***p < 0.001.

## DISCUSSION

The aim of this paper was to examine associations between intergenerational exposure and normative perceptions. As hypothesized, we found an association between parental alcohol use exposure and children's normative perceptions, suggesting parental drinking is a key source of these cognitions in young children. Young children have been shown to hold an extensive array of normative perceptions about alcohol (Cook, Kuntsche, Smit, et al., 2021; Cook, Smit, et al., 2022; Voogt et al., 2020), but thus far, information on the sources of these perceptions has been speculative. Results demonstrate that children who were more frequently exposed to mothers' and fathers' alcohol use attributed alcohol more often to adults depicted in everyday situations in which alcohol use is common. Overall, and as hypothesized, we found exposure to mothers drinking affects children's attributions of alcohol to females and exposure to fathers drinking affects children's attributions of alcohol to the males in the illustration. Moreover, observed patterns held after adjusting for age and sex. Results corroborate previous research showing that parental drinking has an effect on children's normative perceptions (Dalton et al., 2005), specifically by showing that it is not parental drinking but *exposure* to parental drinking which has an effect on their children's alcohol‐related cognitions. Moreover, it extends seminal work by Smit and colleagues on the role of exposure on expectancies (Smit et al., 2018, 2019, 2020), by showing this association with other cognitive dimensions (i.e., here we look at norms rather than expectancies) and among young children (Smit and colleagues looked at adolescents).

The task structure (i.e., depiction of multiple persons) allowed us to examine the robustness of the association between mothers and fathers' exposure and children's normative perceptions across several different people. Results demonstrated that this association held across all adults depicted for exposure to mothers drinking (Table 3). That is exposure affects the attributions of alcohol to all persons of that gender, rather than being dependent on roles (i.e., mother, grandmother, or female friend). This suggests that, already at an early age, children have learned and are making gender‐specific generalizations owing to their exposure to parental alcohol use. Following Bandura's social learning theory (Bandura, 1977) and as hypothesized, these results suggest that it is the gendered characteristics of the drinker which matter for young children's growing normative perceptions. Thus, if mothers consume alcohol more often in situations in which children are exposed to their consumption, then children are more likely to attribute this behavior to other people, but only those of the same sex. Given the primary role mothers play in raising children and the gendered nature of caregiving (Correll et al., 2007), these results may be the result of children spending more time with mothers, but more data particularly on the time spent with females versus males would be needed to confirm these speculations. Exposure to fathers drinking on the other hand only affected children's attributions to the fathers in the illustration. This may be the result of fathers drinking being more common, as we also show, which presents a more specific observational learning opportunity for children, whereby they can observe and internalize more specific details of the drinker (Bandura, 1977).

Fathers were found to report consuming more frequently in situations in which alcohol use is considered normal and in which children could be exposed (Voogt et al., 2020). The effect sizes reported in Table 1 show that these effects are moderate; however, given the social nature of alcohol consumption and the age of the participating children, these moderate effects are not unexpected. Although fathers were found to report consuming more frequently, we found both mothers's and father's alcohol use exposure has an effect on children's perceptions of drinking norms. These results follow social learning theory (Bandura, 1977) and the intergenerational transference of alcohol use behavior (Campbell & Oei, 2010), whereby adults operate as role models for children at these young ages, acting as the primary source of observational learning (including, but not only for, alcohol). The association between exposure to fathers' drinking and perceptions of drinking norms corresponds with literature on other dimensions of alcohol‐related knowledge, with exposure to fathers' alcohol use found to mediate adolescents' alcohol expectancies (Smit et al., 2019, 2020) and young children's expectancies (Kuntsche & Kuntsche, 2018). While the strong association with exposure to mothers' drinking is less often reported in the literature on other cognitions (though see for example, Waddell et al., 2020), there may be several reasons for this finding in our sample. It may be related to the gendered nature of care giving and the associated impact on gender roles within the family. With mothers still taking on the bulk of the care giving duties, opportunities for them to engage in drinking when children are present may be rare (Crompton et al., 2005; Kuntsche et al., 2012). Thus, when they do drink, this behavior stands out to children as different from the norm. This would follow research suggesting children are highly sensitive to changes in the norm when it comes to alcohol and drinking practices (Cook, Kuntsche, Smit, et al., 2021). Additional research with children themselves asking them to describe in their own words and expand upon the answers given in this task are essential for gaining a deeper understandings of the mechanisms behind these findings.

This study furthers the limited literature on parental alcohol use exposure in general and demonstrates the effects of parental alcohol use among a notably young age group. While this is the first time these results have been demonstrated for children this young in the academic literature, they may not be entirely unexpected for parents who have been shown to be aware (to some extent) of their role in teaching and modeling alcohol practices to their children (Cook, Kuntsche, & Pennay, 2021). Parents are also consumers of alcohol in their own right, who value alcohol and drinking as a form of relaxation and “time out” away from being a parent (Cook, Kuntsche, & Pennay 2021; Cook, Pennay, et al., 2022; Emslie et al., 2012). Thus, in thinking about the translation of these results into prevention or health policy, efforts need to be made to inform parents and carers about the association between parental alcohol exposure and children's alcohol‐related cognitions from young age, particularly the fine‐grained nature of this knowledge (see for example Drinken & Drinken, 2020). However, it would also be important to keep in mind the complex lived experiences of parents and the roles alcohol plays in their lives to ensure efforts are not just admonishing parents (i.e., making them feel worse) and thus risking making them less receptive to prevention efforts (Cook, Pennay, et al., 2022; Jayne et al., 2012; Spencer, 2013).

There are several limitations that need to be kept in mind when interpreting these results. First, the absence of diverse family structures (i.e., same sex parents or single parents) limits the inferences that could be made, and it would be a worthy and important goal of future research to examine these results among a more diverse set of population groups and family units. Similarly, these results may not be generalisable beyond the Dutch context and so it would be important to examine whether these results hold in different drinking cultures, for example dry contexts, or low‐ and middle‐income countries. Given that we are not measuring negative consequences from drinking in the current study, future studies may also wish to include a more comprehensive measure of alcohol use including different patterns of consumption (e.g., hazardous drinking, harmful drinking) which may also affect results. Our measure of parental alcohol use also relies on self‐report data which may be subject to social desirability bias. There are a range of potential covariates that we did not collect information on in this study, including parental rules, parent–child relationship and parents own drinking norms, which may affect children's normative perceptions and parents drinking practices (see for example Brody et al., 2000). Despite these limitations, this study is strengthened by the large longitudinal sample and the multi‐informant design with data from both the parent and the child, and with children's data collected using an age‐appropriate assessment tool. It is also strengthened by the inclusion of data from both mothers and fathers allowing us to examine their influence separately.

## CONCLUSION

In conclusion, in examining the impact of exposure to parental drinking on children's perceptions of drinking norms, we found that what children learn from their parents, especially mothers, is gender‐specific. Results presented here contribute to the limited body of work on exposure and knowledge dimensions other than expectancies, and show that exposure can and does influence norms which are crucial for regulating drinking behavior (Voogt et al., 2013). The results provide compelling evidence of the intergenerational transmission of drinking behaviors at very young ages, and because exposure is gender‐specific, it may be an important first steppingstone toward the gendered drinking identities found in the adult population (Van Laar et al., 2021). The effects of this exposure long term and especially when these children begin to drink is of interest for future studies. Finally, these findings add to the growing evidence base of young children's perceptions, knowledge, and awareness of alcohol well before they first consume (Cook, Smit, et al., 2022; Dalton et al., 2005; Kuntsche et al., 2016), all of which have implications for future alcohol use (Zucker et al., 2008).

## FUNDING INFORMATION

This study was funded by a Vidi Grant 452‐13‐003 awarded from the Netherlands Organization for Scientific Research (NWO) to Emmanuel Kuntsche. This work was supported by an Australian Government Research Training Program Scholarship awarded to Megan Cook.

## CONFLICT OF INTEREST

None to declare.

## Supporting information


Tables S1‐S2

## Endnotes

^1^
Mean consumption of more than 14 (females) or 21 (males) standard drinks per week in the past month (Voogt et al., 2013).

^2^
Mean item score of at least 0.53 on the 5‐point scale of the Rutgers Alcohol Problems Index as well as the of more than 14 (girls) and 21 (boys) drinks per week (Voogt et al., 2013).
