# Supplementary material for: Effects of exposure to mother's and father's alcohol use on young children's normative perceptions of alcohol
Source: Alcohol Clin Exp Res. 2022 Sep 16;46(9):1687–94. doi: 10.1111/acer.14902 (PMC9826212; doi:10.1111/acer.14902)
Supplement: Supplementary file 1 — Tables S1‐S2 [file ACER-46-1687-s001.docx]

**Supporting Information**

In supplementary Table 1 we also include grandparents (that is both grandmothers and grandfathers). Grandparents were allocated alcohol 32% of the time.

Table 1. Mothers’ and fathers’ exposure on children’s attributions to the adult males and females in the illustration (Unstandardized regressions coefficients and standard errors in brackets).

|  | **Females** | **Males** | **Mothers** | **Fathers** | **Grandparents** | **Overall** |
| --- | --- | --- | --- | --- | --- | --- |
| **Model 1** | **β (SE)** | **β (SE)** | **β (SE)** | **β (SE)** | **β (SE)** | **β (SE)** |
| **Exposure to Mothers drinking** | .741 (.139)^***^ | -.007 (.148) | .515 (.084)^***^ | .041 (.086) | .037 (.073) | 5.233 (1.097)^***^ |
| **Exposure to Fathers drinking** | .069 (.150) | .453 (.173)^**^ | .034 (.095) | .346 (.099)^***^ | .219 (.087)^*^ | 1.753 (1.148) |
|  |  |  |  |  |  |  |
| **Model 2 controlling for covariates** | |  |  |  |  |  |
| **Exposure to Mothers drinking** | .802 (.141)^***^ | .047 (.149) | .563 (.084)^***^ | .076 (.088) | .058 (.074) |  |
| **Exposure to Fathers drinking** | .027 (.145) | .354 (.174)^*^ | .002 (.091) | .284 (.100)^**^ | .174 (.087)^*^ |  |
| **Sex** | -.357 (.229) | .445 (.254) | -.291 (.140)^*^ | .244 (.148) | .229 (.128) |  |
| **Age** | .376 (.159)^*^ | .626 (.179)^***^ | .296 (.096)^**^ | .396 (.105)^***^ | .271 (.088)^**^ |  |

Sex coded 0=boys, 1=girls.

^⁎^p < .05; ^⁎⁎^p < .01; ^⁎⁎⁎^ p < .001.

Table 2. Mothers’ and fathers’ exposure on children’s attributions to the adult males and females in the illustration with sex and age interaction effects (unstandardized regressions coefficients and standard errors in brackets).

|  | **Persons depicted in the illustrations** | |
| --- | --- | --- |
|  | **Females** | **Males** |
| **Exposure to mothers drinking** | 1.035 (.166)^***^ | -.140 (.207) |
| **Exposure to fathers drinking** | -.096 (.201) | .480 (.269) |
| **Sex** | -.097 (.562) | .339 (.738) |
| **Sex * Fathers Exposure** | .272 (.291) | -.268 (.352) |
| **Sex * Mothers Exposure** | -.521 (.284) | .418 (.291) |
| **Age** | .389 (.158)^*^ | .616 (.178)^**^ |

Sex coded 0=boys, 1=girls.

^⁎^p < .05; ^⁎⁎^p < .01; ^⁎⁎⁎^ p < .001.
